# Fourier analysis of collagen bundle orientation in myocardial infarction scars

**DOI:** 10.1007/s00418-022-02132-x

**Published:** 2022-08-10

**Authors:** Víctor Marcos-Garcés, Cesar Rios-Navarro, Fabián Gómez-Torres, Jose Gavara, Elena de Dios, Ana Diaz, Gema Miñana, Francisco Javier Chorro, Vicente Bodi, Amparo Ruiz-Sauri

**Affiliations:** 1 Department of Cardiology, Hospital Clínico Universitario de Valencia, Valencia, Spain; 2 INCLIVA Health Research Institute, Valencia, Spain; 3 Universidad Industrial de Santander, Escuela de Medicina, Bucaramanga, Colombia; 4 Center for Networked Biomedical Research–Cardiovascular (CIBER-CV), Madrid, Spain; 5 Central Unit for Research in Medicine (UCIM), University of Valencia, Valencia, Spain; 6 Department of Medicine, University of Valencia, Valencia, Spain; 7 Department of Pathology, University of Valencia, Valencia, Spain; 8 Departamento de Patología, Facultad de Medicina y Odontología, Universitat de València, Avda/Blasco Ibáñez nº15, 46010 València, Spain; 9 Department of Cardiology, Hospital Clínico Universitario de Valencia, Valencia, Spain Instituto de Investigación Sanitaria del Hospital Clínico Universitario de Valencia (INCLIVA), Valencia, Spain; 10 Centro de Investigación Biomédica en Red de Enfermedades Cardiovasculares (CIBER-CV), Madrid, Spain; 11 Department of Medicine, Faculty of Medicine and Odontology, University of Valencia, Blasco Ibanez 17, 46010 Valencia, Spain

**Keywords:** Myocardial infarction, Scar, Collagen, Orientation, Fourier

## Abstract

Collagen bundle orientation (CBO) in myocardial infarct scars plays a major role in scar mechanics and complications after infarction. We aim to compare four histopathological methods for CBO measurement in myocardial scarring. Myocardial infarction was induced in 21 pigs by balloon coronary occlusion. Scar samples were obtained at 4 weeks, stained with Masson’s trichrome, Picrosirius red, and Hematoxylin–Eosin (H&E), and photographed using light, polarized light microscopy, and confocal microscopy, respectively. Masson’s trichrome images were also optimized to remove non-collagenous structures. Two observers measured CBO by means of a semi-automated, Fourier analysis protocol. Interrater reliability and comparability between techniques were studied by the intraclass correlation coefficient (ICC) and Bland–Altman (B&A) plots and limits of agreement. Fourier analysis showed an almost perfect interrater reliability for each technique (ICC ≥ 0.95, *p* < 0.001 in all cases). CBO showed more randomly oriented values in Masson’s trichrome and worse comparability with other techniques (ICC vs. Picrosirius red: 0.79 [0.47–0.91], *p* = 0.001; vs. H&E-confocal: 0.70 [0.26–0.88], *p* = 0.005). However, optimized Masson’s trichrome showed almost perfect agreement with Picrosirius red (ICC 0.84 [0.6–0.94], *p* < 0.001) and H&E-confocal (ICC 0.81 [0.54–0.92], *p* < 0.001), as well as these latter techniques between each other (ICC 0.84 [0.60–0.93], *p* < 0.001). In summary, a semi-automated, Fourier-based method can provide highly reproducible CBO measurements in four different histopathological techniques. Masson’s trichrome tends to provide more randomly oriented CBO index values, probably due to non-specific visualization of non-collagenous structures. However, optimization of Masson’s trichrome microphotographs to remove non-collagenous components provides an almost perfect comparability between this technique, Picrosirius red and H&E-confocal.

## Introduction

Despite widespread availability of primary coronary reperfusion and generalized use of myocardial revascularization strategies in acute myocardial infarction, a significant number of patients unfortunately still experience irreversible cardiac damage due to ischemia, ischemia–reperfusion, or both (Heusch and Gersh 2016). In mammals, extensive myocardial injury causing the death of a large number of cardiomyocytes triggers an organized inflammatory and cellular response which leads to the formation of collagen-based scars (Frangogiannis 2014, 2017).

Translational strategies aimed at cardiac protection or regeneration after myocardial infarction have as yet been generally unsuccessful (Heusch and Gersh 2016; Heusch 2020). In this context, there is growing interest in characterizing infarct scars. No longer considered dead tissue, infarct scars encompass extracellular matrix components such as collagen fibers and cells, mainly fibroblasts and myofibroblasts (Frangogiannis 2014, 2017), and active interplay between these elements leads to the final composition and structural and biomechanical characteristics of the infarct scar (Voorhees and Han 2014; Li 2020).

Several infarct scar characteristics, such as collagen bundle orientation (CBO), exert a considerable influence on scar mechanics and undesirable complications after infarction (Voorhees and Han 2014; Richardson et al. 2015). Evidence suggests that CBO and scar anisotropy are major determinants of ventricular function, biomechanical properties, and complications in the post-infarction period (Rouillard and Holmes 2012; Richardson and Holmes 2016; Quinn et al. 2016; Rusu et al. 2019). Specifically, scar heterogeneity may impair systolic function through increased mechanical anisotropy and reduced myocardial strength (Korenczuk et al. 2019). Characterizing collagen bundle alignment and disposition in infarct scars has therefore gained importance in recent years.

One of the most reliable techniques to measure CBO is Fourier analysis, which has been used in collagen-rich conjunctive tissues such as infarct scar (Hervas et al. 2016) and skin reticular dermis (van Zuijlen et al. 2002; Verhaegen et al. 2012b). However, controversy surrounds the best histopathological technique to perform this measurement. Masson’s trichrome (Saeed et al. 2006; Iles et al. 2015), Picrosirius red visualized by polarized light microscopy (Whittaker et al. 1989; Iles et al. 2015; Hervas et al. 2016), and Hematoxylin–Eosin (H&E) visualized by confocal microscopy (Quinn et al. 2016) have all been used to analyze collagen structure in scarred myocardium. Although CBO by Fourier analysis has shown comparable results with these staining and microscopy techniques in the skin reticular dermis (Marcos-Garcés et al. 2017), no head-to-head comparison has been made in infarct scars, so the preferred technique remains currently unknown. Other techniques such as electron microscopy and second harmonic generation imaging have also been used for this purpose but they are technically challenging and their availability is more limited (Kischer and Shetlar 1979; Mostaço-Guidolin et al. 2017).

In our study, we used the scar tissue of a porcine model of acute myocardial infarction to analyze the interrater reliability and comparability of CBO measurement by Fourier analysis in four different methods: Masson’s trichrome, optimized Masson’s trichrome with digital removal of non-collagenous structures, Picrosirius red and polarized light microscopy, and H&E and confocal microscopy.

## Materials and methods

### Experimental study

Twenty-four 25–30 kg juvenile (3 months old) female pigs were used for this study, which was approved by the local Animal Care and Use Committee, and which conforms to the Guide for the Care and Use of Laboratory Animals published by the US National Institutes of Health (NIH Publication No. 85-23, revised 1993) as well as to The ARRIVE guidelines (www.nc3rs.org.uk/ARRIVE). Animals were obtained from the local farm “El Pampo” (registration number: ES462440000003). Three swine with induced myocardial infarction died prior to euthanasia, so they were not considered for analyses. Further details of our study protocol can be consulted elsewhere (Hervas et al. 2016).

Animals were sedated using intra-muscular 8 mg/kg ketamine and 0.1 mg/kg medetomidine. The right jugular vein was cannulated for drug administration and blood sampling purposes. A 10 mg/kg/h continuous intravenous infusion of 2% propofol was used throughout the procedure and pre-treatment with intravenous amiodarone (300 mg), lidocaine (30 mg), and heparin (3000 U) was administered. Pigs were mechanically ventilated using a 50% oxygen gas mixture. Continuous electrocardiographic monitoring of heart rate, rhythm, and ST-segment changes was performed.

A 7F sheath was introduced into the right femoral artery. Invasive blood pressure monitoring was performed. The left anterior descending coronary artery (LAD) was selectively catheterized with a 7F Amplatz Left 0.75 catheter and a standard hydrophilic angioplasty wire was advanced and placed in the distal LAD. A 2.5 × 15-mm angioplasty balloon was inflated at 6 atm in the mid LAD. Coronary artery occlusion was confirmed by contrast injection and by electrocardiographic ST-segment elevation, and was maintained for 90 min, after which the balloon was deflated, and restoration of normal coronary flow was documented by angiography. The animals were then allowed to recover.

After 4 weeks, coronary angiography was repeated, and coronary occlusion or dissection were ruled out. Pigs were euthanized with potassium chloride and hearts were excised. The whole heart was immediately sectioned into 5-mm-thick short-axis slices. Slices were then incubated in a 2,3,5-triphenyltetrazolium chloride (TTZ; Merck Millipore, Billerica, MA, USA) 2% solution for 20 min at 37 °C. Slices were observed under room light and infarcted tissue was defined as the myocardial area that did not stain with TTZ.

### Sample processing and staining

Tissue samples of the infarcted zone were fixed in a 4% formaldehyde solution and embedded in paraffin. Consecutive histological 5-μm-thick sections were obtained from each sample. Masson’s trichrome staining was manually performed using Harris’ Hematoxylin, Biebrich Scarlet/Acid Fuchsin and Aniline Blue as reagents. For Picrosirius red, sections were stained in 0.1% Direct Red 80/Sirius Red F3B (C.I. 35,780, Sigma-Aldrich, St. Louis, MO, USA) in saturated picric acid for 1 h at room temperature, and then sections were differentiated in 0.5% acetic acid prior to dehydration, clearing, and mounting (Junqueira et al. 1979; Osman et al. 2013). The Hematoxylin–Eosin protocol included deparaffinization, alcohol passes, staining in Hematoxylin for 5 min, hydrochloric alcohol 0.5% and ammonium hydroxide 1.5% washes, and staining in Eosin 1.5% for 5 min.

### Light, polarized light, and confocal microscopy imaging

Samples stained with Masson’s trichrome were photographed with a Leica DMD108 light photomicroscope (Leica Microsystems, Wetzlar, Germany). The same photomicroscope was used for Picrosirius red, and polarization was achieved with the Leica Analyzer Module Slider 11,544,002 polarized filter (Leica Microsystems). H&E-stained samples were imaged with the Leica TCS SP2 spectral confocal and multiphoton system (Leica Microsystems). The excitation and detection of eosin was set at 543 and 580–640 nm, respectively. A 10X/NA 0.4 objective was used and images were obtained in a 1024 × 1024 pixel format. A pinhole setting of 1 Airy unit was used to obtain an optical section with an approximate thickness of 5 μm, equal to the whole thickness of the histological sections. Images were adapted to the full dynamic range of the system (8 bits) (Verhaegen et al. 2012b, a).

**Fig. 1:**
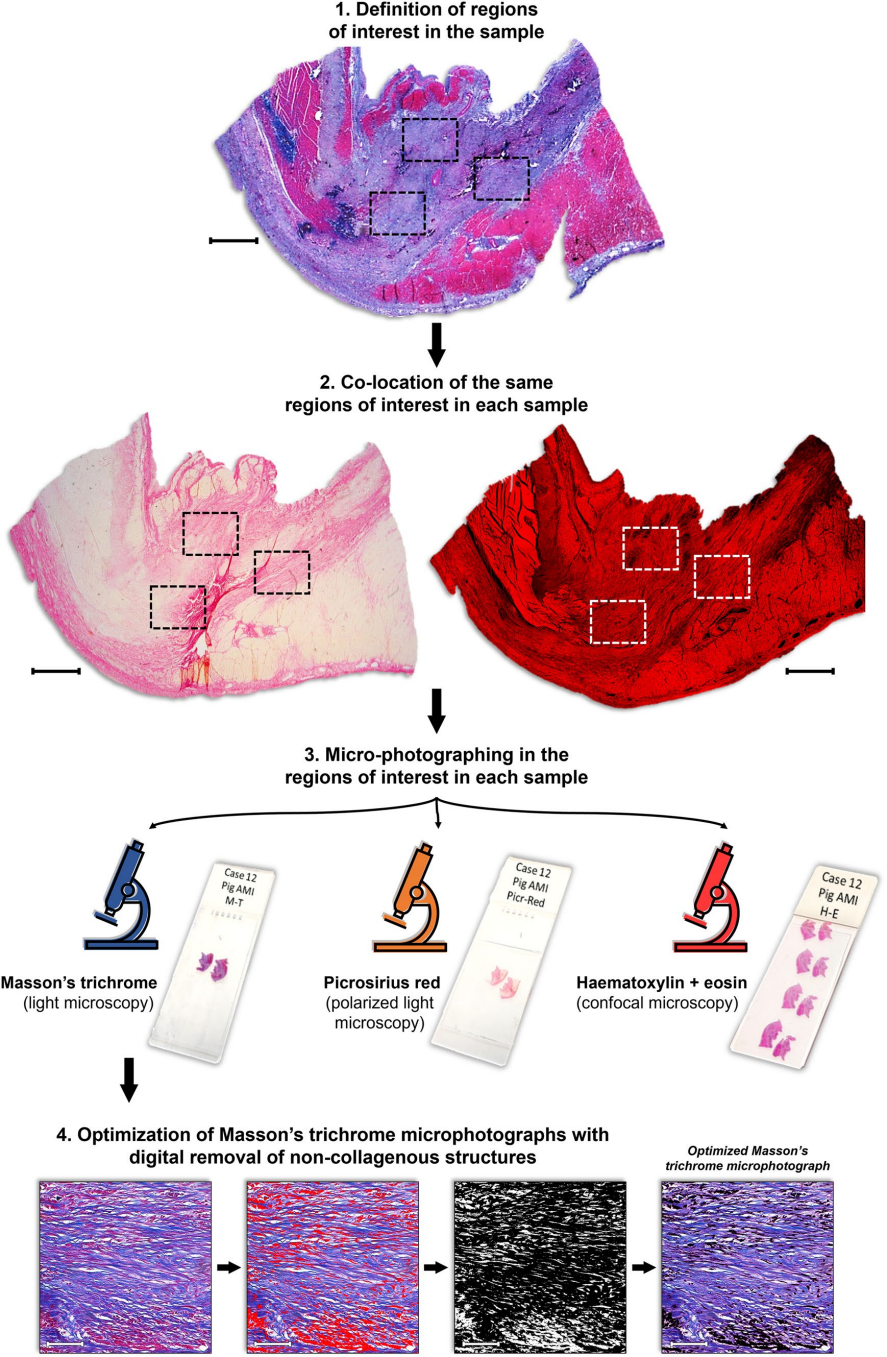
Protocol for microphotograph of regions of interest in infarct scar samples. After sample visualization with light microscopy (H&E and Masson’s trichrome), regions of interest are defined, i.e., in the central area of the infarct scar. For each technique, 21 histological samples and 105 microphotographs (five per sample) were analyzed. Using histopathological references, the same regions are manually co-localized in each sample and microphotographs are taken using the described protocol. Finally, Masson’s trichrome microphotographs were optimized with digital removal of non-collagenous structures. Note that the Picrosirius red image (*center left*) depicts a macro photograph without polarized light microscopy. This macro caption as well as the Masson’s trichrome (*upper*) one have been edited for brightness and contrast to increase understandability.* Bar* (macro images) = 1 mm; *bar* (microphotographs) = 100 µm. *H&E* Hematoxylin–Eosin

Sample visualization with light microscopy was performed before microphotography for quality control. An automated scan of the whole sample was performed in confocal microscopy to select regions of interest (in the central core of infarcted myocardium), and five microphotographs with a 200 × magnification were taken from these areas. Next, these same regions were manually co-located in Masson’s trichrome and Picrosirius red samples and five microphotographs were obtained for each case and stain. The microscopy and imaging protocol is summarized in the flow diagram in Fig. 1.

### Optimized Masson’s trichrome microphotographs

Masson’s trichrome microphotographs were optimized to minimize the influence of non-collagenous structures in CBO measurement. For this instance, segmentation was performed to select cellular structures (stained in purple), a segmentation mask was obtained, and this segmentation mask was subtracted from the original microphotograph. In the resulting image, only collagenous components remained to be incorporated in CBO measurement. The process is summarized in Fig. 1.

### Collagen bundle orientation by Fourier analysis

CBO was measured by Fourier analysis in each microphotograph, using the Image-Pro Plus 7.0 image analysis software (Media Cybernetics, Silver Spring, MD, USA). Briefly, the collagen orientation index is the ratio of the maximum width (minor axis) and the maximum length (major axis) of the thresholded Fourier 2D power plot (van Zuijlen et al. 2002), ranging from 0 (indicating more parallel oriented collagen bundles) to 1 (indicating more randomly distributed collagen bundles).

**Fig. 2:**
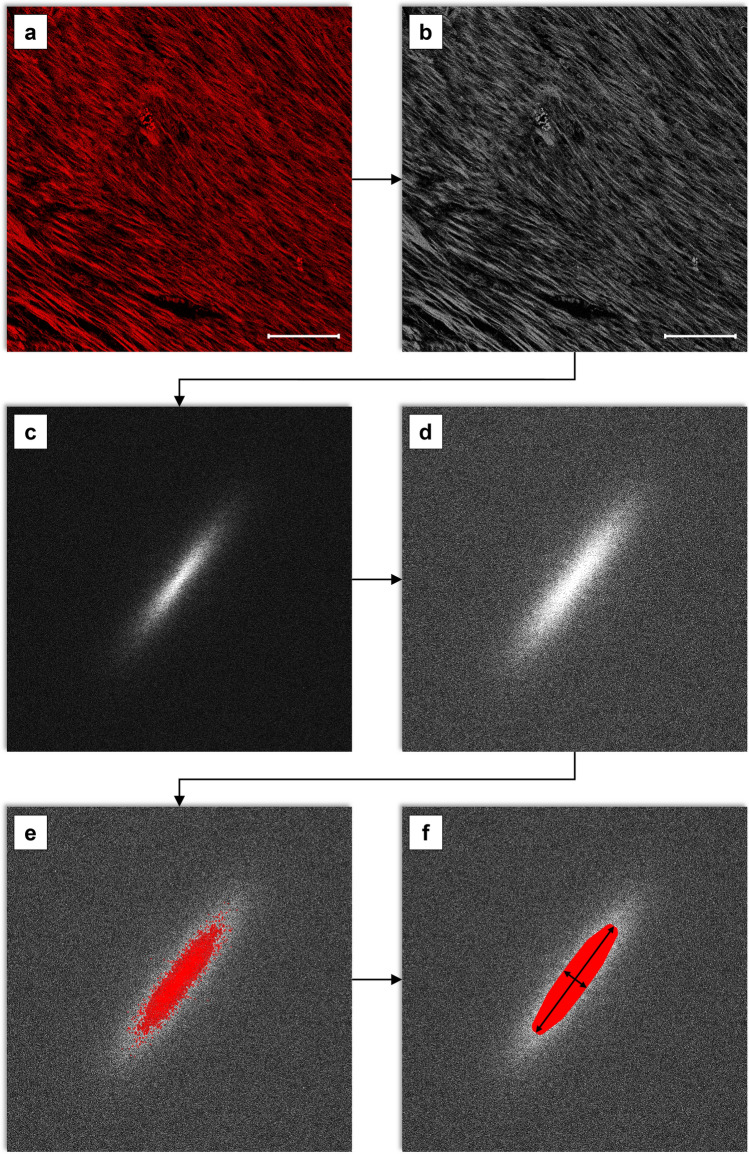
Protocol for CBO index measurement by Fourier analysis. **a** Microphotograph of a pig myocardial scar sample stained with H&E and visualized with confocal microscopy. A Best Fit filter has been applied. **b** Conversion to grey scale 16. **c** FFT has been applied to the image and the 2D power plot has been obtained. **d** The spectrum gain has been adjusted to obtain a more defined power plot. **e** Manual segmentation to select the central frequencies in the FFT power plot. **f** Automatic major and minor axis measurement after implementing an area filter range and Smoothing, Pre-Filter, and Convex Hull filters.* bar* = 100 µm. For each technique, 21 histological samples and 105 microphotographs (five per sample) were analyzed. *CBO* collagen bundle orientation, *FFT* fast Fourier transform. *H&E* Hematoxylin–Eosin

Two independent researchers blindly and independently performed the measurements for all samples. A macro program was developed to automate the process (Fig. 2). In each microphotograph, the following steps were applied: (1) A Best Fit filter was applied to maximize contrast between collagen bundles and background. (2) The image was converted to grey scale 16. (3) The fast Fourier transform (FFT) was applied, obtaining a 2D power plot of the image. Half FFT and the Log. Scale spectrum was used, and the spectrum gain was adjusted (between 70 and 200) in some instances to obtain a more defined power plot. (4) Manual segmentation in the 0 to 255 scale was performed to select the central frequencies in the power plot. (5) Small objects were excluded from measurement by means of an area filter range. (6) Holes were filled and Smoothing, Pre-Filter, and Convex Hull filters were applied to obtain a homogeneous plot. (7) Manual correction with the Split objects tool was required in some cases to exclude spikes in the plot. (8) Automatic maximum length and width (major and minor axis) of the segmented plot were obtained.

The protocol was semi-automated. Human intervention was limited to adjusting the spectrum gain of the FFT and excluding spikes in the power plot in some cases, and manual segmentation of the power plot in every case.

For each sample, measurements of the five microphotographs were averaged to calculate the mean collagen orientation index.

### Statistical analysis

Histograms were computed for non-averaged CBO measurements with the four different techniques.

Reliability analysis was performed using the intraclass correlation coefficient (ICC), Bland–Altman plots and limits of agreement, and coefficient of variation (CV). The two-way random effect model for consistency and average measures was selected to calculate ICCs and their respective 95% confidence intervals.

The Bland–Altman plots and limits of agreement (Bland and Altman 1986) were calculated by plotting the differences between two sets of measurements against the mean value obtained from both sets of measurements, then calculating the mean and standard deviation of the differences between the measurements, and finally depicting the limits of agreement with a 95% confidence interval, i.e., the mean plus or minus two standard deviations of the mean difference between the measurements.

The coefficient of variation (CV) (Shechtman 2013) was calculated using the following formula: CV (%) = [(SE_meas_/mean) × 100], where SE_meas_ is the standard error of the measurements. Lower values in this statistical measure of reliability and consistency between two sets of measurements indicate higher consistency between measurements.

Two reliability analyses were performed. First, interrater reliability (reproducibility) between two different observers was analyzed for CBO measurements in each technique, to determine which technique allows for more reproducible measurements between different observers. We next assessed consistency of CBO measurements between Masson's trichrome, optimized Masson’s trichrome, Picrosirius red, and H&E-confocal techniques, to confirm whether CBO could be equally measured in each technique.

Statistical significance was achieved at two-tailed *p* < 0.05. The SPSS statistical package version 22.0 (SPSS Inc., Chicago, IL, USA) was used for analysis.

## Results

### Interrater reliability (reproducibility) of CBO index by Fourier analysis

Using the previously described semi-automated, Fourier-based method, two different observers measured the CBO index in the same microphotographs. Measurements were compared to analyze interrater reliability.

**Fig. 3:**
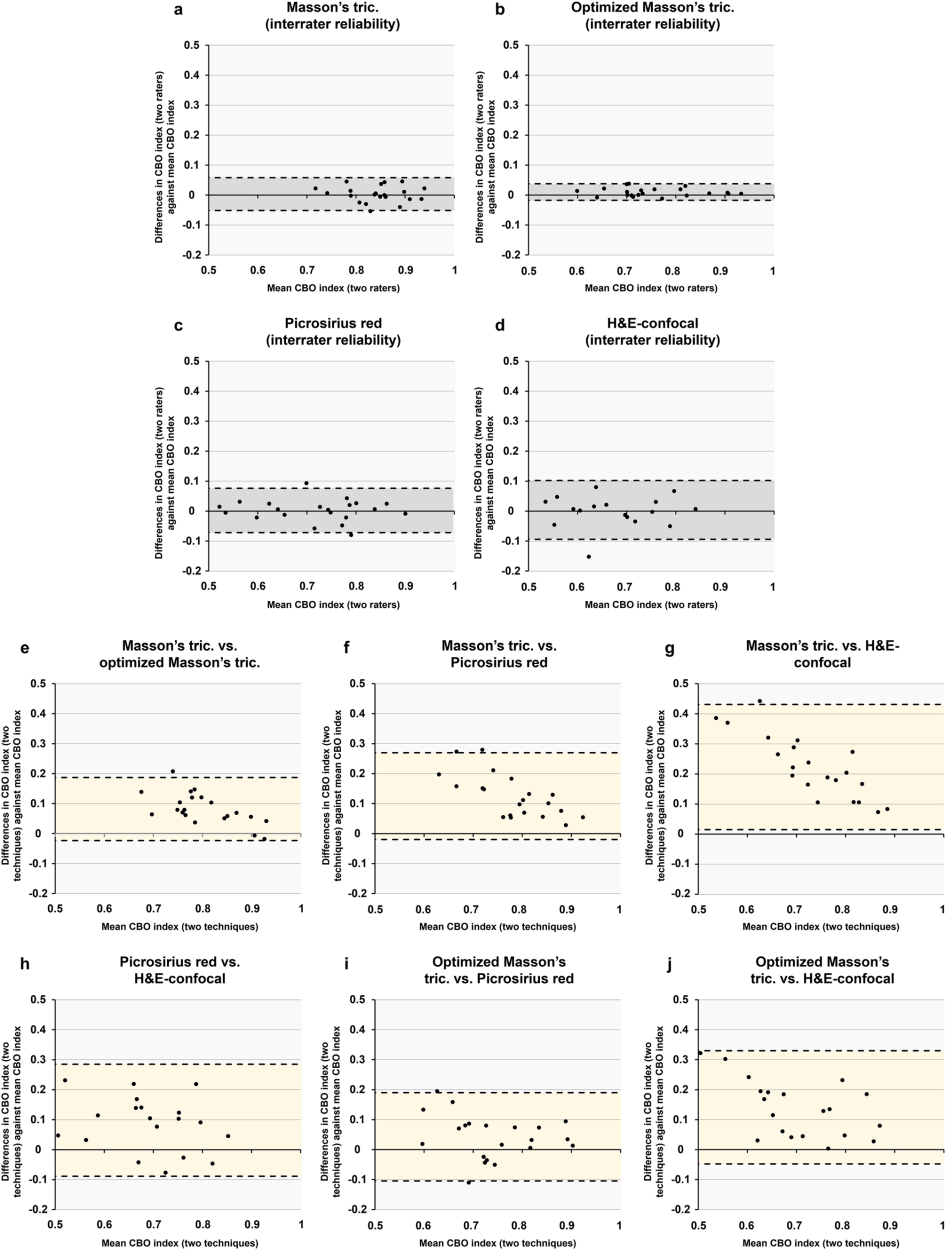
Bland–Altman plots and limits of agreement for CBO index measurement by Fourier analysis. In each plot, the average collagen orientation index as measured by the two different methods being compared is presented in the *x*-axis, and differences in collagen orientation index measurements between these two methods are plotted in the *y*-axis. Limits of agreement are graphically represented (as* dashed lines* and* shadowed area*) and calculated as mean ± 2 SDs of the mean difference between the measurements. For each technique, 21 histological samples and 105 microphotographs (five per sample) were analyzed. **a**–**d** Plots for interrater reliability (reproducibility) of measurements in each technique. **e**–**j** Plots for consistency between staining and microscopy techniques. *CBO*  collagen bundle orientation, *SDs* standard deviations

Agreement between two observers was almost perfect in all techniques (Masson’s trichrome, optimized Masson’s trichrome, Picrosirius red, and H&E-confocal), as shown by ICC analysis (0.95, 0.99, 0.97, and 0.97, respectively) and narrow Bland–Altman limits of agreement (Table 1 and Fig. 3a–d), thus revealing an almost perfect reproducibility of CBO index measurements by this method for each staining and microscopy technique.

**Table 1:** Interrater reliability (reproducibility) of CBO index measurement by Fourier analysis

	Masson’s trichrome	Optimized Masson’s trichrome	Picrosirius red	H&E-confocal
ICC (95% CI)	0.95 (0.87–0.98)	0.99 (0.99–1)	0.97 (0.93–0.99)	0.97 (0.93–0.99)
*p* value for ICC	< 0.001	< 0.001	< 0.001	< 0.001
Bland–Altman limits of agreement	0.06, − 0.05	0.04, − 0.02	0.08, − 0.07	0.10, − 0.09
%CV (SE_meas_)	7.03 (0.06)	12.07 (0.09)	14.88 (0.11)	23.07 (0.14)

*CBO* collagen bundle orientation, *ICC* intraclass correlation coefficient, *CV* coefficient of variation, *SE*_*meas*_ standard error of measurement *CI* confidence interval

### Consistency of CBO index between different techniques

We tested the agreement of CBO index between Masson’s trichrome, optimized Masson’s trichrome, Picrosirius red, and H&E-confocal as measured by one observer.

**Fig. 4:**
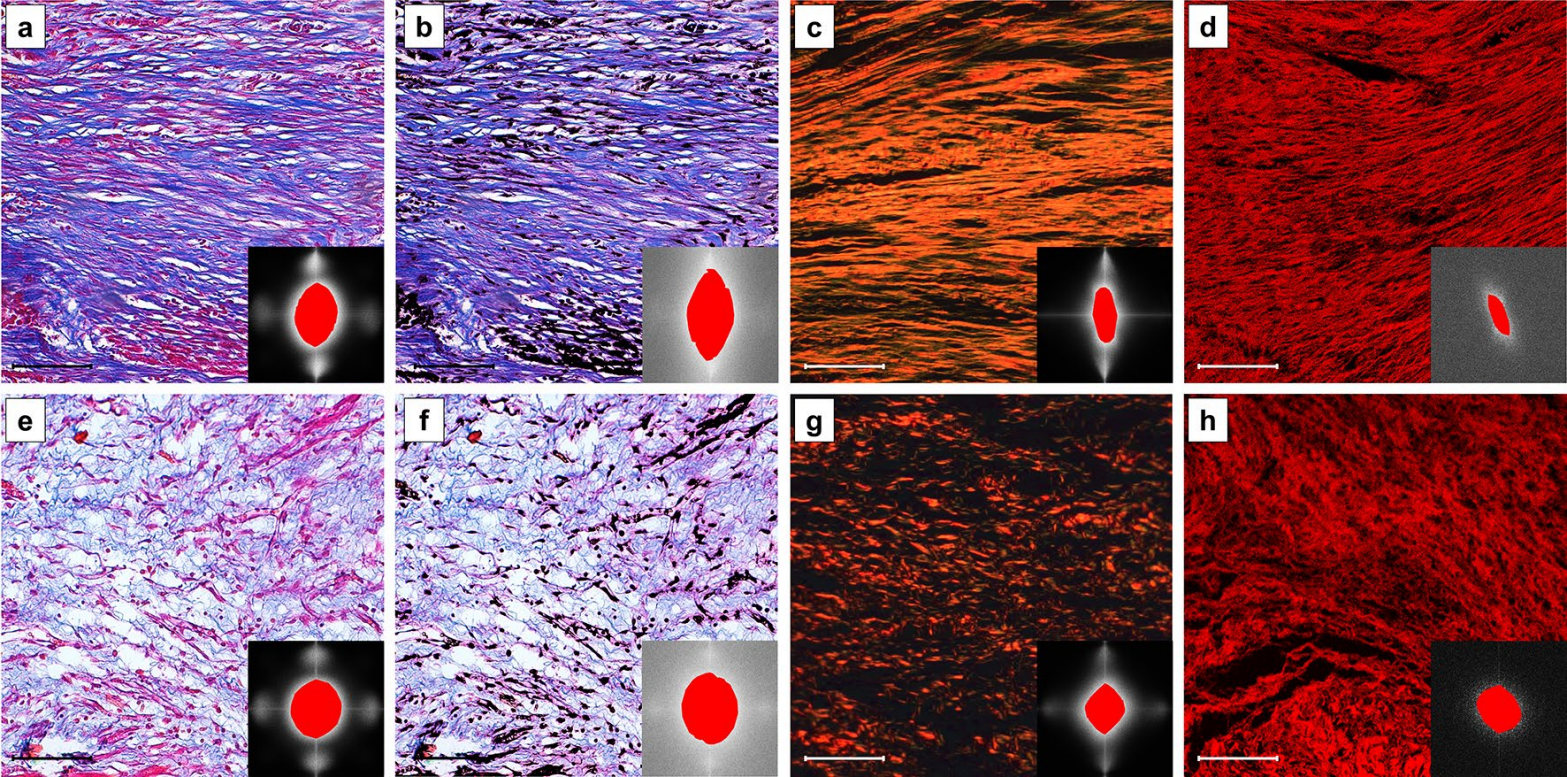
Examples of CBO index in infarct scarring with each staining technique. Samples were stained with Masson’s trichrome (visualized with light microscopy, **a** and **e**), optimized Masson’s trichrome with digital removal of non-collagenous structures (**b** and **f**), Picrosirius red (visualized with polarized light microscopy, **c** and **g**) and H&E (visualized with confocal microscopy, **d** and **h**). In the first row (**a**–**d**), a case of parallel-oriented collagen bundles is shown. Collagen orientation index for each image is 0.67, 0.52, 0.41, and 0.35. In the second row (**e**–**h**), a case of randomly oriented collagen bundles is depicted. Collagen orientation index for each image is 0.88, 0.8, 0.82, and 0.82.* bar* = 100 µm. For each technique, 21 histological samples and 105 microphotographs (five per sample) were analyzed. *CBO* collagen bundle orientation, *H&E* Hematoxylin–Eosin

Consistency was almost perfect between Masson’s trichrome and optimized Masson’s trichrome (ICC 0.87 [0.67–0.95], *p* < 0.001), optimized Masson’s trichrome and Picrosirius red (ICC 0.84 [0.6–0.94], *p* < 0.001), optimized Masson’s trichrome and H&E-confocal (ICC 0.81 [0.54–0.92], *p* < 0.001), and Picrosirius red and H&E-confocal (ICC 0.84 [0.60–0.93], *p* < 0.001). However, consistency was strong between Masson’s trichrome and Picrosirius red (ICC 0.79 [0.47–0.91], *p* = 0.001) and moderate between Masson’s trichrome and H&E-confocal (ICC 0.70 [0.26–0.88], *p* = 0.005) (Table 2). Bland–Altman limits of agreement were consistent with ICC (Fig. 3,e–j). An example of microphotographs and CBO index measurement in each case is provided in Fig. 4.

**Table 2:** Consistency for CBO index measurement by Fourier analysis between different techniques

	Masson’s trichrome vs. optimized Masson’s trichrome	Masson’s trichrome vs. Picrosirius red	Optimized Masson’s trichrome vs. Picrosirius red	Masson’s trichrome vs. H&E-confocal	Optimized Masson’s trichrome vs. H&E-confocal	Picrosirius red vs. H&E-confocal
ICC (95% CI)	0.87 (0.67–0.95)	0.79 (0.47–0.91)	0.84 (0.6–0.94)	0.70 (0.26–0.88)	0.81 (0.54–0.92)	0.84 (0.60–0.93)
*p* value for ICC	< 0.001	0.001	< 0.001	0.005	< 0.001	< 0.001
Bland–Altman limits of agreement	0.19, − 0.02	0.27, − 0.02	0.19, − 0.1	0.43, 0.01	0.33, − 0.04	0.28, − 0.09
%CV (SEmeas)	8.95 (0.07)	13.54 (0.11)	12.39 (0.09)	21.23 (0.16)	15.79 (0.11)	19.88 (0.13)

*CBO* collagen bundle orientation, *ICC* intraclass correlation coefficient, *CV* coefficient of variation, *SEmeas* standard error of measurement, *CI* confidence interval

### Distribution of CBO index measurements in each technique

**Fig. 5:**
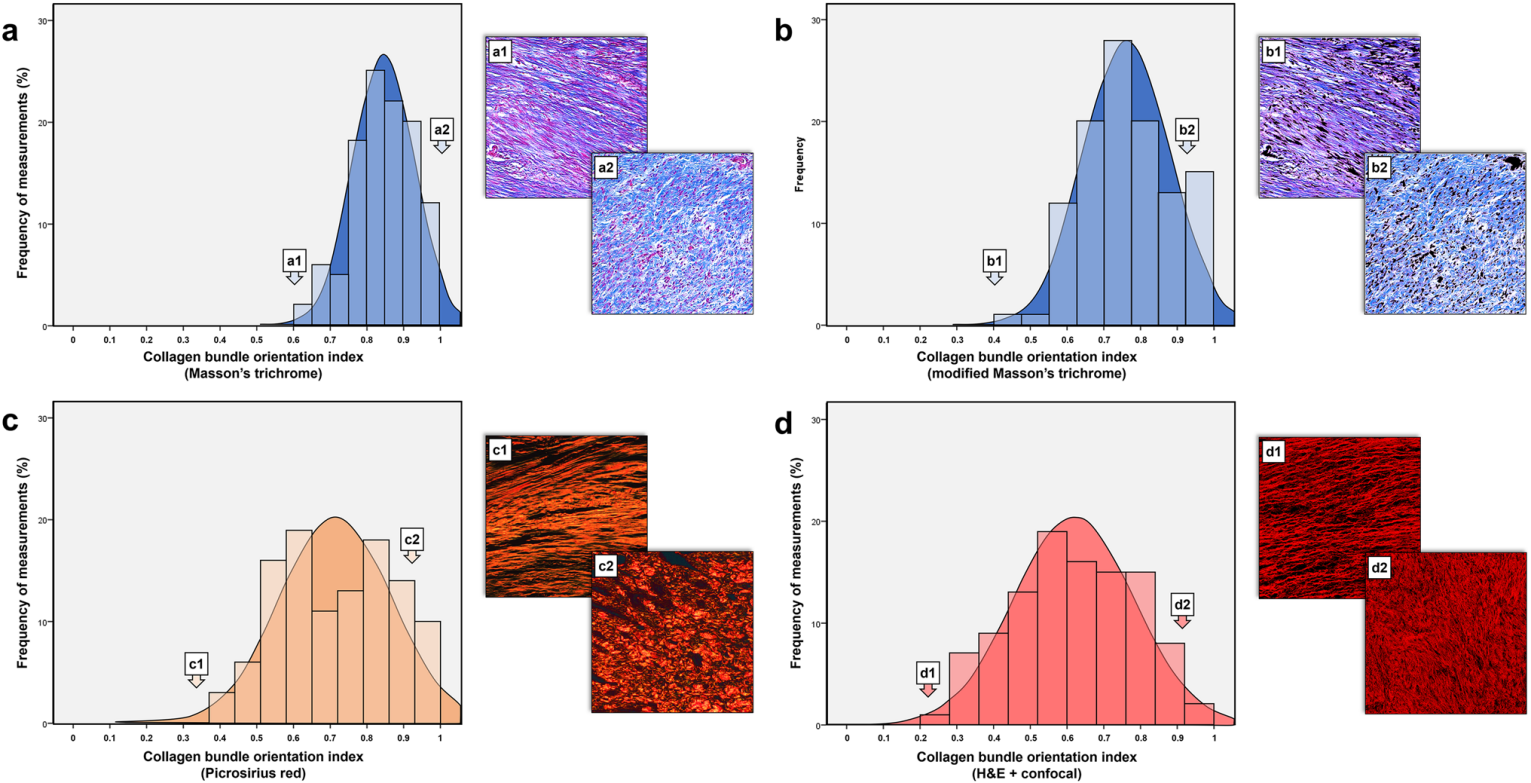
Histogram of CBO index measurements in the different techniques. **a** Histogram in Masson’s trichrome and examples of a parallel (a1) and random (a2) CBO index pattern in myocardial scar. Bin width: 0.05. Bin centers: 0.61, 0.67, 0.74, 0.79, 0.83, 0.88, 0.92 and 0.97. **b** Histogram in optimized Masson’s trichrome with digital removal of non-collagenous structures and examples of a parallel (b1) and random (b2) CBO index pattern in myocardial scar. Bin width: 0.075. Bin centers: 0.47, 0.49, 0.6, 0.66, 0.74, 0.82, 0.89 and 0.95. **c** Histogram in Picrosirius red and examples of a parallel (c1) and random (c2) CBO index pattern in myocardial scar. Bin width: 0.07. Bin centers: 0.4, 0.48, 0.55, 0.61, 0.69, 0.76, 0.82, 0.89 and 0.95. **d** Histogram in H&E + confocal and examples of a parallel (d1) and random (d2) CBO index pattern in myocardial scar. Bin width: 0.08. Bin centers: 0.28, 0.33, 0.4, 0.49, 0.57, 0.63, 0.73, 0.8, 0.87 and 0.93. For each technique, 21 histological samples and 105 microphotographs (five per sample) were analyzed. *CBO* collagen bundle orientation, *H&E* Hematoxylin and Eosin

Histograms of all individual measurements of CBO index for each technique are depicted in Fig. 5. In Masson's trichrome, CBO index measurements tended towards more randomly oriented values (closer to 1). Distribution of measurements was also narrower, as shown in the histogram and depicted in the lower CV for both interrater reliability (7.03%, Table 1) and consistency between Masson’s trichrome and other techniques (13.53% and 21.23%, Table 2).

In contrast, in Picrosirius red and H&E-confocal measurements were more widely distributed between parallel oriented (closer to 0) and randomly oriented (closer to 1) values, and more parallel oriented values were noted, especially in H&E-confocal. This wider distribution of values is consistent with the higher CV for both interrater reliability (14.88 and 23.07%, Table 1) and consistency between techniques (Table 2).

However, compared to Masson’s trichrome, optimized Masson’s trichrome also depicts more widely distributed values (CV 12.07%, Table 1), thus identifying more parallel-oriented cases.

The differential distribution of measurements in each technique can explain the almost-perfect consistency between optimized Masson’s trichrome and the other techniques and Picrosirius red and H&E-confocal, since they provide widely distributed and more parallel-oriented CBO values. It also explains the lower consistency between Masson's trichrome and Picrosirius red and H&E-confocal, since this tends to provide more narrowly distributed and randomly oriented CBO values.

## Discussion

In our study, we prove that a semi-automated, Fourier-based image analysis method can provide highly reproducible measurements of CBO in four different histopathological techniques, namely Masson’s trichrome, optimized Masson’s trichrome, Picrosirius red and polarized light microscopy, and H&E and confocal microscopy. Masson’s trichrome tends to provide more randomly oriented CBO index values, probably due to non-selective staining of collagen bundles, and its comparability with Picrosirius red and H&E-confocal is worse. Nonetheless, an almost perfect agreement is noted between optimized Masson’s trichrome, Picrosirius red, and H&E-confocal regarding the CBO index.

### Infarct scarring

Nowadays, many patients experiencing acute myocardial infarction develop some extent of myocardial scarring due to irreversible cardiac damage and the death of a variable amount of cardiomyocytes (Heusch and Gersh 2016). Myocardial ischemia due to coronary blood flow impairment, generally due to a thrombotic occlusion of an epicardial coronary artery, triggers a cascade of events defined as the inflammatory, proliferative, and maturation phases (Prabhu and Frangogiannis 2016). Some animals, such as adult zebrafish, are capable of completely regenerating a healthy myocardium even after extensive cardiac injury (Ryan et al. 2020; Ross Stewart et al. 2021). However, due to the limited regeneration capability of mammal myocardial tissue, the sudden death of a large number of cardiomyocytes triggers an organized inflammatory and cellular response which eventually leads to formation of collagen-based scarring (Frangogiannis 2014, 2017).

Despite other promising cardiac protection strategies beyond reperfusion, translational attempts have so far been unsuccessful at limiting infarct size or improving outcomes in patients with acute myocardial infarction (Heusch and Gersh 2016; Heusch 2020). In this setting, scar formation seems like the lesser evil, given the lethal consequences of improper scar development such as cardiac rupture. Interest is therefore growing in infarct scarring, which is no longer considered dead tissue, but rather a dynamic interplay of extracellular matrix components (mainly collagen fibers) and cells (mainly fibroblasts and myofibroblasts) (Rog-Zielinska et al. 2016).

### Scar mechanics and post-infarction complications

Being the most predominant component of infarct scars, the role of collagen is central. A balance should ideally exist between synthesis and degradation of extracellular matrix components, and inadequate deposition of scar components and impaired collagen assembly can lead to wall thinning, aneurysm formation, and eventually cardiac rupture or heart failure (Ma et al. 2014). Conversely, excessive collagen accumulation and extensive scarring correlates with increased wall stiffness and development of diastolic dysfunction (Moreo et al. 2009; Wang et al. 2020). However, controlled collagen cleavage is required for efficient scar formation (Nong et al. 2011).

Beyond collagen synthesis and deposition, CBO and other organizational parameters impact considerably on scar mechanics and undesirable post-infarction complications such as left ventricular dilation, wall rupture, and heart failure (Zimmerman et al. 2000; Fomovsky et al. 2012; Voorhees and Han 2014; Ma et al. 2014; Richardson and Holmes 2016; Gabriel-Costa 2018).

The relationship between collagen bundle structure and ventricular mechanics is bidirectional: not only can collagen architecture influence the biomechanical properties of the left ventricle, but these mechanical characteristics can also impact the structure of collagen bundles in infarcted scars (Zimmerman et al. 2000; Fomovsky et al. 2012). For instance, it has been shown that collagen bundle organization can regulate scar fibroblast fate and function (Seo et al. 2020; Bugg et al. 2020). Aligned collagen disposition induces differentiation to a myofibroblast phenotype, which more actively remodels the scar extracellular matrix. Myofibroblasts and other cells tend to generate collagen and other extracellular matrix components parallel to their own tissue orientation, which reinforces the two-way relationship between collagen bundle alignment and biomechanical scar properties (Ma et al. 2014; Lin et al. 2020).

### Techniques for scar collagen characterization

The complexity of infarct scar mechanics, tissue architecture, and post-infarct complications highlights the need for strategies to accurately characterize the disposition of scar components.

Several non-invasive methods for infarct scar characterization have been developed (Quinn et al. 2016; Perez-Terol et al. 2019), but histopathological analysis of excised samples is the most reliable procedure. Aside from considerations such as the most desirable animal model, method for myocardial infarction induction, timing of scar analysis, and image analysis method for measurement, investigators must decide which histological staining and microscopy techniques are to be performed in the samples.

Several techniques have been used to analyze collagen structure in scarred myocardium, such as Masson’s trichrome (Saeed et al. 2006; Iles et al. 2015), Picrosirius red visualized by polarized light microscopy (Whittaker et al. 1989; Iles et al. 2015; Hervas et al. 2016), and Hematoxylin–Eosin (H&E) visualized by confocal microscopy (Quinn et al. 2016). Masson’s trichrome is an inexpensive, easily performed stain which can be visualized with standard light microscopy. However, it provides non-specific results, staining collagen bundles, but also cellular components. Unlike this former method, Picrosirius red and H&E (visualized with confocal microscopy) provide specific staining and/or visualization of collagen, excluding non-collagenous components from the performed measurements. Nevertheless, these latter techniques are associated with increased cost and technical complexity, since Picrosirius red requires polarized light microscopy for collagen bundle visualization, and in H&E, confocal microscopy is needed to select the specific excitation and detection of Eosin, the colorant that stains the collagen bundles (Verhaegen et al. 2012b, a). On the other hand, other available techniques such as electron microscopy and second harmonic generation imaging are even more technically challenging (Kischer and Shetlar 1979; Mostaço-Guidolin et al. 2017).

### Comparison of techniques to measure CBO

In our study, we focus on a specific parameter of collagen bundle architecture in infarct scars, namely CBO, and a specific method for performing this measurement, Fourier analysis. Fourier analysis has previously been used to study CBO in collagen-rich conjunctive tissues such as the infarct scar (Hervas et al. 2016) and the skin reticular dermis (van Zuijlen et al. 2002; Verhaegen et al. 2012b). Although CBO by Fourier analysis has been shown to be comparable with Masson’s trichrome, Picrosirius red and H&E-confocal staining, and microscopy techniques in the skin reticular dermis (Marcos-Garcés et al. 2017), ours is the first study to perform a head-to-head comparison in infarct scarring.

First, we show that a semi-automated method based on Fourier analysis can provide highly reproducible CBO indices in four different techniques. Objective histopathological measurements are essential to obtain accurate and reproducible results in laboratory studies (Baak 2002; Laurinavicius et al. 2012), and this is provided by our method regarding CBO measurement in infarct scarring.

Nonetheless, we prove that significant differences exist when CBO index is analyzed in different histopathological techniques. Picrosirius red and H&E-confocal are comparable and show almost perfect agreement. Both Picrosirius red and H&E-confocal allow for specific visualization of collagen bundles (Bancroft and Layton 2013), which could explain their high intertechnique comparability. Interestingly, measurement distribution was similar across the two techniques. CBO index values were distributed from more parallel-oriented (closer to 0) to more randomly oriented (closer to 1) values. With these techniques, therefore, CBO by Fourier analysis can accurately differentiate cases with more parallel-oriented collagen bundles and cases with more randomly oriented collagen bundles.

However, intertechnique comparability was worse when Masson’s trichrome was compared against Picrosirius red and H&E-confocal. Measurements of CBO index in Masson’s trichrome tended to group around more randomly oriented values (closer to 1), indicating lower discriminative ability in cases with more parallel-oriented values. It should be noted that Masson’s trichrome, while commonly performed to analyze scars and collagen-rich structures, is not specific to collagen bundle visualization, and also stains other structures such as cells, albeit with a different colorization (Bancroft and Layton 2013). Since in Fourier analysis all image components are incorporated into the 2D power plot which is then used for CBO index measurement (van Zuijlen et al. 2002), we hypothesize that including non-collagen structures in this staining can alter the measurement, specifically towards more randomly oriented values.

To support this hypothesis, we performed an optimization of Masson’s trichrome microphotographs by digitally subtracting the non-collagenous components. Comparability of CBO in optimized Masson’s trichrome was almost perfect with Picrosirius red and H&E-confocal. In this case, CBO values were also more widely distributed, and the technique was able to identify more parallel-oriented cases. Even though the “true” CBO cannot be ascertained, the almost-perfect agreement between these techniques implies that they could be performing equally well to measure this parameter.

In a previous study by our group, we showed that CBO index measurement in skin reticular dermis is comparable to Fourier analysis using Masson’s trichrome, Picrosirius red, and H&E-confocal (Marcos-Garcés et al. 2017). Nonetheless, the skin reticular dermis is a dense connective tissue with a clear predominance of collagen bundles (Kanitakis 2002), and cellular elements represent only a marginal area of the histological microphotographs (Marcos-Garcés et al. 2017), while in our 4-week infarct scar pig model, cellular components were relatively frequent in the histological microphotographs. Thus, it could be theorized that optimization of Masson’s trichrome microphotographs should only be needed if the non-collagenous components fill a relevant area of the images.

In summary, we provide evidence of a highly reproducible, Fourier-based method for CBO measurement in an animal model of infarct scarring. Masson’s trichrome samples prove to be less comparable with Picrosirius red and H&E-confocal, probably due to non-specific collagen staining, but optimization of Masson’s trichrome images provides an almost-perfect comparability with the other techniques. Given the current need for protocol standardization of animal models of myocardial infarction (Richardson et al. 2015; Lindsey et al. 2021), our study represents an advance in the selection of histopathological techniques and measurement methods for collagen bundle characterization in infarct scarring.

### Study limitations

Among the limitations of the study, despite similarities in the pathophysiological process of myocardial infarction and infarct scar composition between humans and pigs, caution should be exercised when translating our results to humans. Second, myocardial scar development is a dynamic process; we provide evidence of CBO measurement comparability at 4 weeks after infarction, but this comparability may vary at different times after infarction depending on scar maturation. Third, co-location of samples for microphotographs was performed manually, yet automatic co-location of samples might have provided more head-to-head and direct comparisons between techniques. Lastly, other techniques that are routinely used in the assessment of the extracellular matrix structure, such as second harmonic generation imaging or electron microscopy, should have been performed to provide a more comprehensive comparison. However, the study was initially designed to include only standard histochemistry techniques. Further studies should explore this topic.

## Conclusions

A semi-automated, Fourier-based image analysis method can provide highly reproducible CBO measurement in four different histopathological techniques, namely Masson’s trichrome, optimized Masson’s trichrome, Picrosirius red and polarized light microscopy, and H&E and confocal microscopy. Masson’s trichrome tends to provide more randomly oriented CBO index values, probably due to non-specific staining and visualization of non-collagenous structures. However, optimization of Masson’s trichrome microphotographs to remove non-collagenous components provides an almost-perfect comparability between this technique, Picrosirius red and H&E-confocal.
